# A Multispecialty Approach to the Identification and Diagnosis of Nonaccidental Trauma in Children

**DOI:** 10.7759/cureus.27276

**Published:** 2022-07-26

**Authors:** Muhammad Romail Manan, Sara Rahman, Leah Komer, Hamna Manan, Saadia Iftikhar

**Affiliations:** 1 Medicine, Services Institute of Medical Sciences, Lahore, PAK; 2 Basic Sciences, Services Institute of Medical Sciences, Lahore, PAK; 3 Psychiatry, University of Toronto, Toronto, CAN; 4 Medicine, Jinnah Hospital, Lahore, PAK; 5 Radiology, Dr. Samir Abbas Hospital, Jeddah, SAU

**Keywords:** shaken baby syndrome, abusive head trauma, child maltreatment, non-accidental trauma, child abuse and neglect

## Abstract

Child abuse is a preventable phenomenon of considerable concern resulting in significant child mortality and morbidity. We analyze various abuse lesions such as radiological (visceral and skeletal lesions and those associated with head trauma) and cutaneous (burns, bruises, bites, etc.) to enhance streamlined identification of injuries in cases of physical child abuse. For effective results, it is essential to remain mindful of all background factors, such as the caregiver setting and the prevalence of child maltreatment in the concerned community while acknowledging the possibility of natural causes (genetic diseases such as osteogenesis imperfecta and hemophilia, or acquired abnormalities) that can mimic NAT and cause confusion in diagnosis and treatment. The margin of error in cases of abuse is negligible, therefore, making its diagnosis a momentous as well as challenging clinical task. An ineffective diagnosis can have detrimental emotional consequences for the family and may even expose the child to future potentially fatal episodes of abuse. Hence, there is a need to direct special focus on the importance of accurate history taking and immediate, responsible reporting to authorities, as well as to child protective services. Therefore, considering the multifactorial approach this subject requires, this review aims to delve into prevalence statistics, various risk factors, and their effect on psychological health to offer a near-complete regulation to ensure an effective understanding of NAT on part of doctors, social workers, and other relevant authorities.

## Introduction and background

Ambulatory children can suffer various injuries as a result of fairly foreseeable minor accidents that may occur during activities of routine play, therefore, highlighting a considerable need to distinguish these accidental wounds as well as several medical conditions and certain cultural healing practices from the traumatic injuries sustained during deliberate or neglectful acts of assault. Purposefully inflicted injuries, regardless of the intention to cause harm, are considered “Nonaccidental trauma (NAT).” Child maltreatment is categorized either by the performance of a deliberate harmful act on a child or by the failure to ensure safety at the hands of a caregiver. NAT is documented to have the second greatest prevalence amongst the causes of pediatric mortality and is also reported to have an incidence that ranges from 0.47 to 2,000 per 100,000 [1]. These figures depicting NAT incidence are speculated to be largely underreported on accounts of misdiagnosis and unfamiliarity associated with abusive physical injuries [1].

Child abuse contributes to significant pediatric mortality and morbidity as it often goes unidentified until a serious injury has been inflicted [2]. A study reports that up to half of the physically battered children presented to an orthopedic surgeon, and 35% of these patients suffered through multiple occurrences of abuse with 10% of these victims meeting lethal consequences [3]. Additionally, the results of a retrospective cohort study have reported that at least 25% of the children with suspected NAT experienced a recurring episode within one year of initial suspicion [4]. Higher mortality rates have been reported in children who experience repeated events of physical abuse [5]. Such an understanding should call attention to the consequences of recurrent episodes of NAT, and appropriate measures should therefore be set in place to prevent these incidents. Apt identification, effective follow-up, and timely interventions are crucial factors to prevent recurrent episodes of abuse and connecting children and their families to adequate support.

In a retrospective, descriptive chart review, Thorpe et al. concluded that one-third of the pediatric patients, with nonaccidental fractures in various phases of healing, previously presented at least once to a clinical setting owing to signs indicative of traumatic insults, and these prior medical visits manifested as missed opportunities to diagnose abuse [6]. It is essential that medical professionals from various specialties working with children function together to ensure effective documentation of the clinical history and physical examination, imaging studies, and consulting one another for an expert opinion. Additionally, they need to work alongside allied health professionals and social service departments to safeguard the well-being of children. Educating physicians in this regard may help instill a sense of confidence to recognize, diagnose, and report potential victims of abuse. Therefore, this review aims to organize and present the existing literature on pediatric NAT while also discussing risk factors, clinical lesions, investigative techniques, and physical and psychological impacts in order to provide relevant insight to healthcare professionals regarding its identification, and diagnosis.

## Review

Risk factors

There are well-studied risks concerning childhood abuse, and physician awareness of such factors can help identify children and families at an elevated risk. It is essential to correlate the knowledge of these risk factors to appropriate interventions while assessing the risk for future episodes. This can prove helpful for not only screening and identifying abuse, but also connecting at-risk families with adequate support to prevent potential harm.

Factors Associated With the Victim

Risk factors intrinsically concerned with the child which increase their risk of being abused may include age, race, gender, and health status [7]. Age is a prominent risk factor, and it has been found that infants are potentially at a greater risk of experiencing violent physical abuse; therefore, non-ambulatory children, especially those under two years of age enduring fractures, should raise a greater suspicion of abuse when presenting with suspicious injuries [8,9]. Accordingly, it has been recommended that all children under one year of age presenting with fractures should be reported to child protective services [10]. It is worth noting that the susceptibility of being exposed to NAT appears to be independent of the victim’s gender [11]; however, females under the age of 18 are more vulnerable to sexual abuse as compared to males [12].

A study conducted by Zhao et al., using the Kids’ Inpatient Database (KID), demonstrates that children belonging to black ethnic groups and the female gender had a higher probability of being diagnosed with abuse when presenting with fractures [13]. Patients presenting with spinal cord injuries and fractures of the skull were less frequently associated with nonaccidental circumstances as compared to the children presenting with rib fractures [13]. Ultimately, their analysis revealed five predictor variables for the presence of abuse; age, race, burns, fractures of the rib, and intracranial injuries. There is conflicting evidence regarding the association of low birth weight and pre-term birth with child abuse [14]. Initial studies reported an association between the two but Leventhal [15], questioned the validity of these studies based on flawed methodologies. The results of a retrospective cohort study conducted in West Sussex, England reported an increased probability of child protection registration for low birth weight and preterm fetuses. This relationship was found to be unassociated with maternal age and socioeconomic status [14]. Furthermore, the risk of abuse has been studied to be 3.4 times higher in children with disabilities as compared to the risk documented in their fellow non-disabled children [16]. In short, a common pattern is highlighted in the risks intrinsic to the victim; children with heightened health needs, those with special care requirements, and with developmental milestones not in line with parental expectations are generally perceived as difficult to care for and hence, are subject to a heightened risk of maltreatment [17].

It should be noted that crying as a potential stimulus for physical abuse has been recognized in several studies and perpetrator accounts [18]. Anger toward an inconsolable crying infant indicates extreme frustration when positive caregiver efforts fail to bring solace to the infant. Recent understanding highlights the preventable nature of child abuse with crying recognized as a noteworthy stimulus in the recommendations of the American Academy of Pediatrics, and the Canadian Joint Statement on traumatic head injury due to child maltreatment (THI-CM) [18]. Furthermore, targeted, hospital-based, education programs have proven to substantially reduce nonaccidental head injuries in newborns [19].

Factors Related to the Family and Community

Some of the strongest risks associated with the occurrence of child abuse are factors independent of the victim. A lower degree of family cohesion and a higher degree of conflicts within the family are associated with a greater risk for physical abuse [20]. Similarly, parental aggression is another independent risk factor for child maltreatment [20]. The quality of the neighborhood may appear to influence the prevalence of child maltreatment; however, it is worth mentioning that evidence exists to suggest against this perceived correlation [21,22].

Poverty in the neighborhood, and the violent crime rate, on the other hand, do pose as noteworthy determinants of child maltreatment [21,23]. Additionally, communities with a greater immigrant concentration may have a lower incidence of “Parent-to-child physical aggression (PCPA)” [24]. This correlation may be attributed to strong support networks acting as protective factors within these communities with high ethnic diversity and greater immigrant concentration [25]. However, among refugee families, the status of their immigration is highlighted as the most significant risk factor associated with child maltreatment. Several factors notably influenced by the immigration status and add to stress within the family include financial constraints, social isolation, and unemployment [25]. Discriminatory practices and postmigration stressors further aggravate the degree of parental distress giving rise to abusive behaviors, often masked as disciplinary actions to protect the children against external dangers of the host community [25].

Alcohol and drug use in the community also appears to be prominent risk factors, and populations with higher availability of alcohol and a greater degree of drug possession may be linked to an amplified hazard for child abuse events [26]. Differential association between the availability of alcohol and the type of maltreatment experienced by the child has also been reported. Physical abuse is more common in populations containing off-premise alcohol sources, and areas containing a greater number of establishments such as pubs and bars are more closely associated with incidents of neglect [26]. There are also reports that areas with higher rates of opioid prescriptions are linked to an increase in cases of child abuse [27].

Recent COVID-19 restrictions and isolation protocols may have influenced the incidence of family violence including child abuse [28]. Abusers tend to isolate their victims, and COVID-19 restrictions could result in situations where victims are confined within an isolated environment with their abuser. A rise in the incidence of traumatic head injury due to child maltreatment/abusive head trauma (THI-CM/AHT) has been reported by Sidpra et al. in a study conducted in the United Kingdom; therefore, this necessitates the development and progression of appropriate support measures for these victims as COVID-19 restrictions are eased off [29]. In contrast, several child protective services have also documented a decrease in reports of child maltreatment during the pandemic, but this drop may be attributed to decreased opportunities to detect abuse, and it may not accurately represent an actual fall in the incidence of child maltreatment cases [28].

Factors Related to the Abuser

Some of the commonly observed antecedents of abuse, concerning the characteristics associated with the abuser, are troubled childhood, low self-esteem, and unplanned pregnancies [30]. Moreover, financial hardships, poor impulse control, and mental health problems are also considered to be several risk factors influencing the occurrence of child abuse events [31-33]. Parental substance use disorders, mental health conditions, alcoholism, and violence experienced through current romantic partners have all been documented to escalate the risk of child maltreatment. It has been reported that the perpetrator of physical abuse is usually female, but incidents perpetrated by males are more likely to result in a lethal outcome for the victim [34-36]. Additionally, the pattern of abuse is documented to be transmitted across generations, i.e., people abused as children may be more prone to becoming abusive parents themselves [37]. As suggested by recent studies, such transmission of abusive behavior across generations can be prevented by a nurturing relationship with a romantic partner. Family support unrelated to the romantic partner may have the same protective role against the progression of abusive behaviors.

Hence, after discussing the risk determining factors of NAT, it is quite clearly indicated that child abuse is not just a consequence of one element rather it is a multifactorial phenomenon that demands to be dealt with by a detailed analysis of its various dimensions.

Clinical history

Clinical evaluation of the suspected victim should begin with a detailed clinical history. Information on the onset, duration, progression, associated symptoms, and aggravating circumstances should be documented along with an explicit account of the circumstances of the injury. The primary outcome of detailed history in cases of child abuse should be to evaluate support systems and potential risk factors, allowing the caregiver to communicate concerns and feelings, assess the circumstances of the injury, uncovering any history indicative of acquired or congenital mimickers of abuse, and assessing the degree of child’s safety. Victims of child abuse may present with nonspecific symptoms but in the case where a severe injury is sustained, the child may be unresponsive when brought to the clinical setting [38]. Such patients may not effectively be able to convey the circumstances associated with their injury [39]; therefore, caregiver questioning is important in these situations.

**Figure 1:**
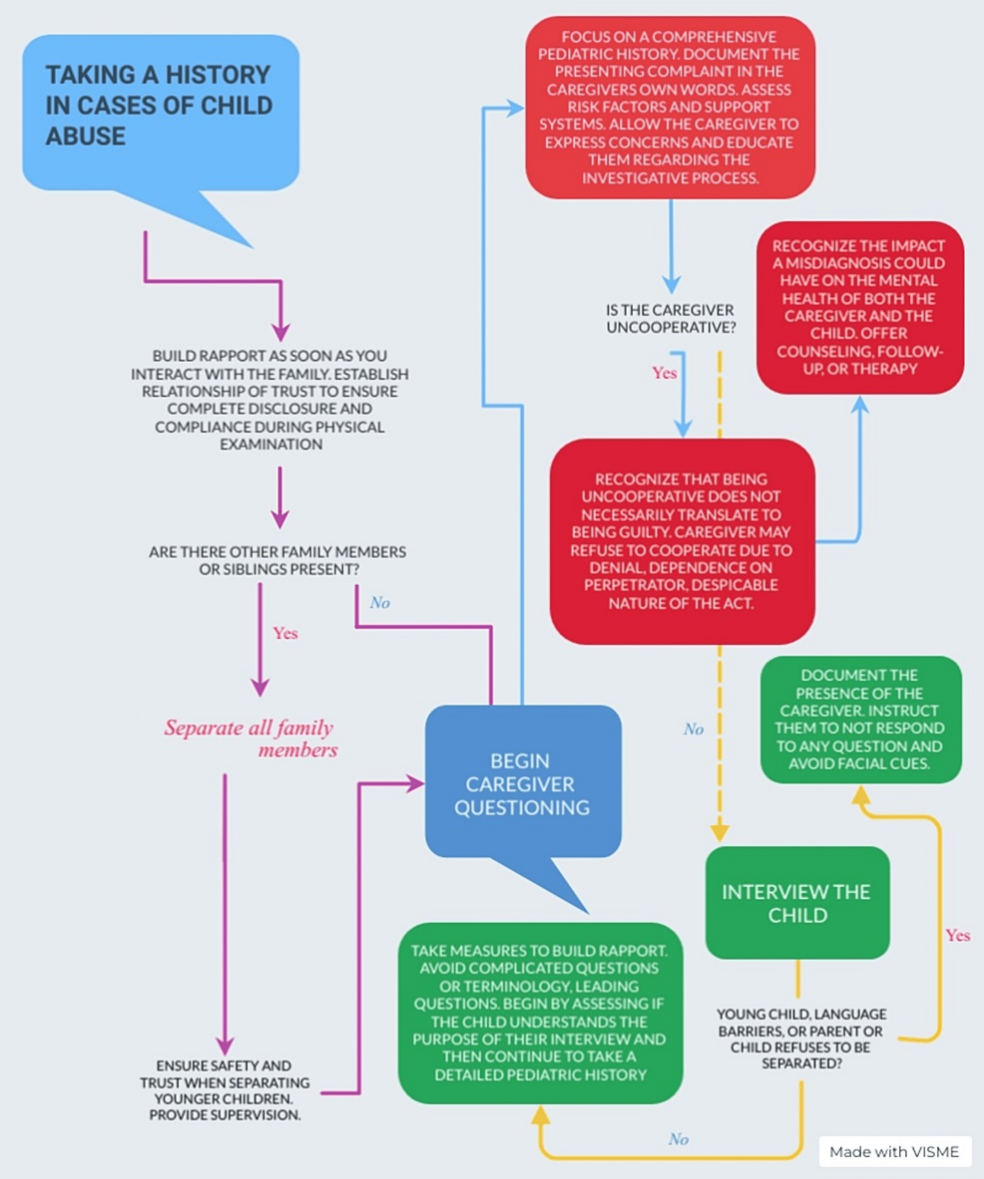
History taking in cases of pediatric physical abuse

While caregiver questioning is an important aspect of history taking in cases of abuse, it is equally crucial to be sensitive to the situation, and therefore, an accusatorial tone must be avoided. Additionally, if the child can communicate clear and comprehensible history, they should be interviewed separately from the caretakers. A sociodemographic profile including names, genders, dates of birth, home, and work addresses, and contact information of all concerning personnel should be recorded meticulously, followed by the medical history. The chief complaint should be recorded in the caregiver’s own words, this serves to later evaluate the circumstances against the account provided by the caregiver. Causes and aggravating factors should be documented along with a detailed history of past hospitalizations, past medical/surgical history, history of allergies, family history of bleeding/bruising, inherited disorders, and history of violence in the family The caregiver should additionally be inquired about potential substance abuse, financial pressures, and circumstances surrounding the pregnancy [38]. A detailed developmental history of the child as well as a history of medical disorders in the family could also possibly assist in uncovering various genetic and metabolic disorders to reduce the chances of misdiagnoses [38]. History of prior clinical visits is equally indicated in determining an abusive etiology since it can provide a thorough insight into a potentially repetitive clusters of injuries. Suspicion surrounding a case of abuse should escalate when the caregiver provides an inconsistent and vague history or delays therapeutic measures significantly [38]. In addition to the aforementioned guidelines for history taking in these cases, several other practice points have been highlighted in Figure 1 [40].

Cutaneous manifestations 

Various cutaneous manifestations of abuse discussed in this review are burns, bite marks, and blunt force injuries. Cutaneous lesions are the most prevalent manifestations of nonaccidental trauma, and it is estimated that up to 90% of the victims presenting with suspected abuse have various injuries to the skin [41]. Family physicians, pediatricians, emergency room doctors, and other healthcare professionals are distinctively equipped with the ability to appropriately diagnose various cutaneous presentations of abusive injuries, while, dermatologists play a significant role in identifying mimickers of abuse. Therefore, healthcare professionals have a significant role in diagnosing and possibly preventing child maltreatment by appropriately documenting and reporting written and photographic records of the case.

Bruises

**Figure 2:**
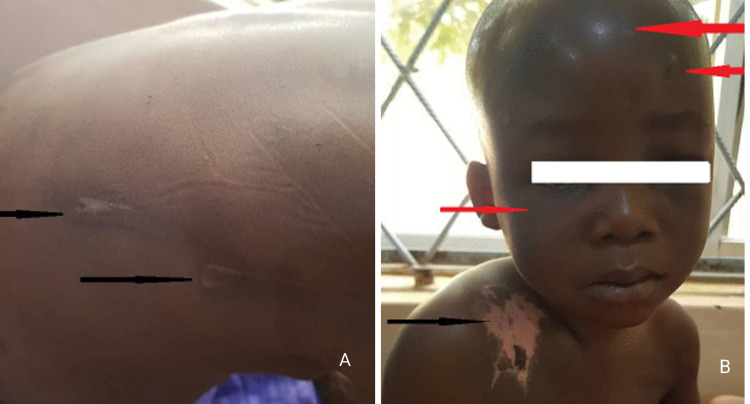
A four-year-old boy with battered child syndrome (A) Scars and rope marks over the back. (B) Forehead swelling, bruises, darkish discoloration around the lower part of the eyes (Panda eyes sign) (red arrows) and burns on the right shoulder (black arrow) [48]. Case courtesy of Ibrahim et al. [48]. (Creative Commons Attribution License)

Bruising is the most commonly reported cutaneous lesion associated with child maltreatment which is oftentimes the first sign of abuse with healthcare professionals being aware of such minor injuries in 41.9% of the cases before any fatal trauma has occurred [42,43]. “Those who don’t cruise, rarely bruise.”, a cross-sectional study conducted by Sugar et al. highlighted the understanding that ambulatory children bruise more commonly while, bruising in children under the age of nine months should raise suspicion of an abusive or pathologic etiology [44]; therefore, it is crucial to evaluate bruising within the context of the child’s ambulation. Additionally, factors such as size, location, shape, and pattern of the bruise should be keenly observed to differentiate accidental injury from nonaccidental. Bruising over bony prominences is more suggestive of an accidental event while bruising over areas padded with fat, such as the face, buttocks, earlobes, neck, philtrum, and genital region is more frequently connected to an abusive etiology [45]. Additionally, bruises of smaller size, with undefined borders, are indicative of accidental bruising; moreover, healthcare professionals should remain vigilant of the phenomenon of patterned bruising. Accidental bruises generally are not patterned but inflicted bruises may retain the shape of the object used to cause the trauma e.g., the pattern of a hand [46]. Other patterns such as loop and crescentic streaks are also concerning for NAT along with easily recognizable patterns such as those resulting from belts or hangers [45]. Tramline pattern of bruising may suggest that the trauma was inflicted by means of a blunt, rod-shaped instrument [47], and bruises from the strike of a rope [48], may present with abrasions scattered over the bruised skin (Figure 2A) [48]. Therefore, a detailed description of the injury along with associated integumental disruptions should be recorded to further verify and deduce the nature of the circumstances and causative mechanisms.

It is also worth noting, however, that bruise patterns observed in gluteal and petechial bruising along the rim of the pinna depend on the morphology of the body part and not necessarily on the shape of the object used to inflict the injury, therefore, a distinct pattern may not be evident in such cases [47]. Moreover, accidental bruising is more commonly seen on the shins and knees, and owing to the immobility of the child, bruises on the head and face of pre-ambulatory children are uncommon but such bruising can be observed as a consequence of minor accidents in toddlers [49]. In short, factors such as ambulatory status of the child, location of the bruise, number of bruises with a mean of 2.4 bruises per accidentally injured walking child [44], and bruise clusters as well as petechiae are common in cases of nonaccidental trauma [50], thus can facilitate in differentiating accidental bruise from nonaccidental.

**Figure 3:**
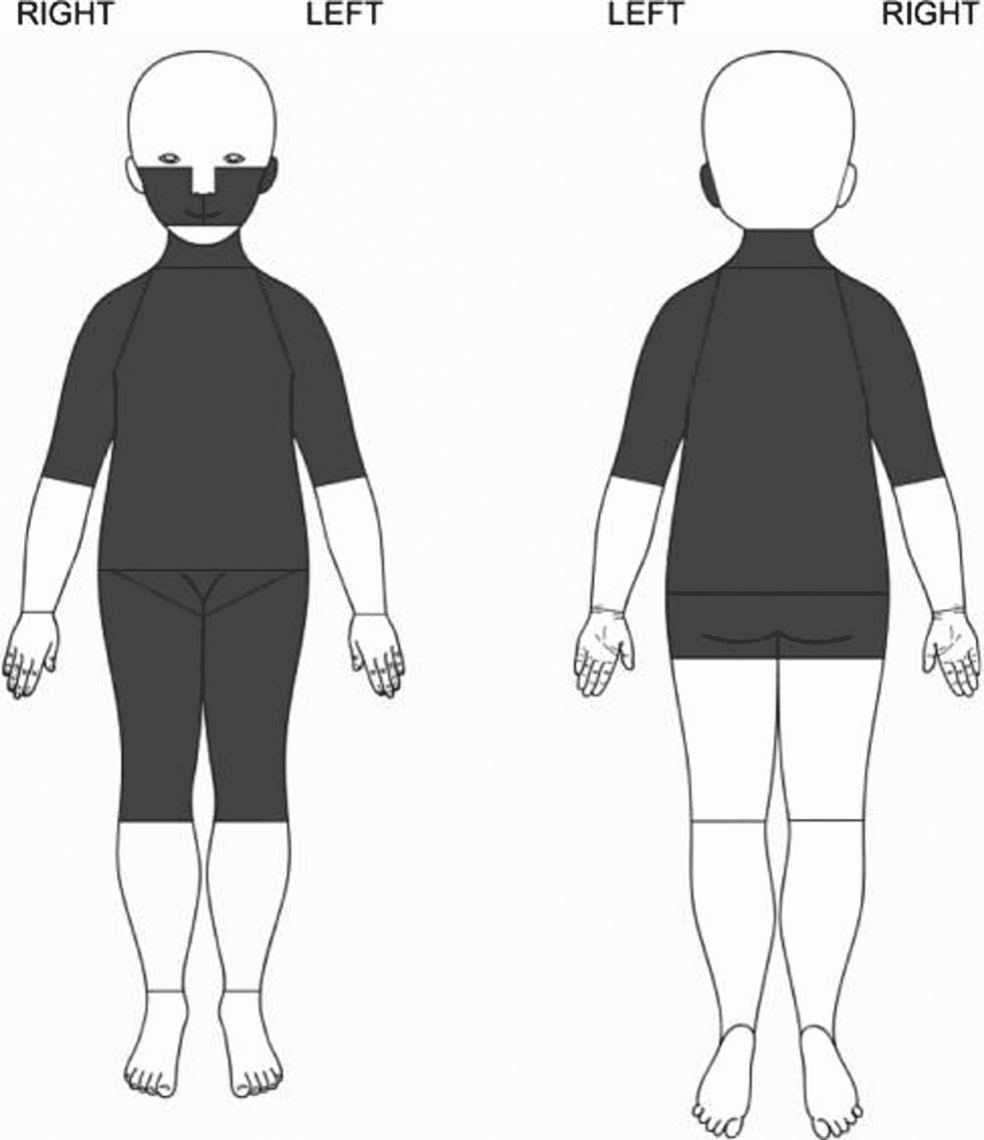
Bruises on highlighted areas were more likely associated with cases of those who was physically abused than non-abused Case courtesy Kemp et al. [50]. (Creative Commons Attribution Non-Commercial)

Color was previously used to document the age of the bruise, however, as per the Canadian Paediatric Society position statements and practice points, this understanding has been rendered unreliable in a clinical setting due to variability in healing patterns [51]. It should be noted that none of the above-mentioned characteristics are absolutely indicative of an abusive etiology but some locations, patterns, circumstances, and aspects of bruise raise a greater suspicion of abuse than others. Hence, healthcare professionals should be vigilant of all suspicious injuries. To highlight the red flags associated with bruising, Pierce et al. developed a bruising clinical decision rule indicating highly abuse-specific locations of the body in an infant younger than four years [52]. The rule is the TEN-4, which stands for torso, ear, neck, and bruising in an infant younger than four months. This rule has been since updated to include the frenulum, angle of the jaw, cheeks, eyelids, and subconjunctiva and infants under 4.99 months (TEN-4-FACESp) of abuse. Bruising present in any of the TEN-4-FACESp regions is highly indicative of abuse in children younger than four years. Additionally, Kemp et al. [50] have pictorially represented (Figure 3) the results of their retrospective cross-sectional study highlighting particular bruise locations in children more likely to be associated with cases of confirmed physical abuse in comparison to cases where abuse was ruled out.

**Figure 4:**
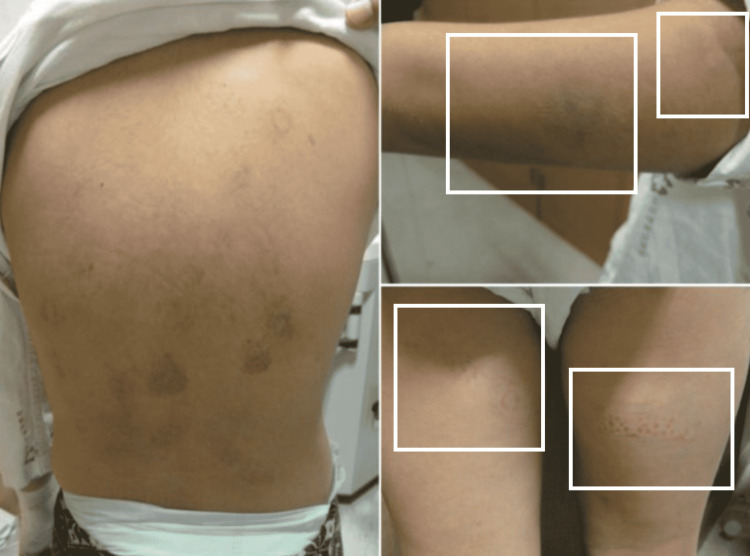
Various sizes of bruises on the back, arm, and thigh of a nine-year-old girl Case courtesy of Joo and Kim [53]. (Creative Commons Attribution Non-Commercial License)

Some forms of child abuse such as dilutional hyponatremia resulting from the ingestion of excessive quantities of water enforced by the perpetrator may also be associated with other unapparent cutaneous lesions such as bruises (Figure 4) [53]; therefore, while recording and reporting abuse in a child there should be an emphasis on a detailed description of the cutaneous lesion along with a thorough physical examination including general body parameters, and examination of the hands, feet, trunk, genitalia, mouth, nares, and earlobes.

Burns

Pediatric burns may result as a consequence of three major etiologies; accidents, negligent caretaking, and nonaccidental trauma [54]. 1.7% to 25% of the pediatric patients presenting with burns have a nonaccidental etiology [55]. This wide variation in prevalence may exist due to the dissimilarity in the definitions of nonaccidental trauma, or the physician’s hesitancy in diagnosing abuse [55]; abusive burns (Figure 2B), however, are still frequently reported among children aged <3 years [56]. A 12-point criteria are described below to substantiate the nonaccidental nature of pediatric burns in cases of suspected abuse, and healthcare professionals should be mindful of these red flags when managing suspected victims of abusive burns [57].

1) Absence of eyewitnesses

2) Injury declared to be inflicted by other children

3) Relatives bringing the child to the healthcare setting

4) Exceedingly withdrawn child

5) Symmetrical and full thickness burns (even on hands and feet)

6) Isolated burns on the buttocks or the perineum

7) Numerous scars or hematomas in different stages of healing

8) Other injuries indicating maltreatment

9) Past clinical visits with a history of trauma

10) Delay in seeking medical attention

11) Inconsistent history of time since injury

12) Injury not corresponding with a degree of child’s development

Abusive burn lesions may manifest as dry thermal injuries, wet burns (scalds), or patterned burns. Scalds are the most frequently detected lesion in patients reporting abusive burns [58]. Scald injuries are divided into two types, spill and splash burns, and immersion burns. Spill and splash injuries appear as scalds with variable penetrating depth and irregular margins [59]. The lesions of the accidental and nonaccidental spill and splash injuries appear clinically identical and hence, these injuries pose a significant challenge in the diagnosis of abuse [59]. However, these lesions are frequently seen in cases of accidental trauma and when not associated with an abusive etiology may typically be located on the head and back [60]. To the point, scalding in the form of spill and splash lesion present on the buttocks and the perineum should always raise suspicion of abuse [61]. Owing to the difficulty associated with connecting spill and splash injuries to abusive intent, it is important to be suspicious of all such injuries that do not positively correspond with the degree of the child’s ambulation, the extent of their development, and clinical history.

The second type of scalded burn injury is caused by submersing the child into a hot liquid. Forced immersion burns appear symmetrical with distinctly marked borders called tide marks [62]. Tide marks serve as indications of the air-water interface and assist in the estimation of the degree of immersion. Additionally, unlike spill and splash injuries, the scalds caused by immersion are associated with a uniform thickness throughout the lesion. Moreover, three characteristic features are observed in cases of forced immersion injuries [61]. Glove and stocking pattern of scalded burns is observed when the hands or feet of the child are forcibly submerged in the hot water. Pattern termed as “Zebra stripes” appears when the creases of the body are spared due to a flexed bodily position. A donut hole sparing pattern is commonly seen when the buttocks of the immersed child are pressed against the surface of the bathtub. The bathtub, being cooler than the water, prevents scalding from developing on the buttocks.

**Figure 5:**
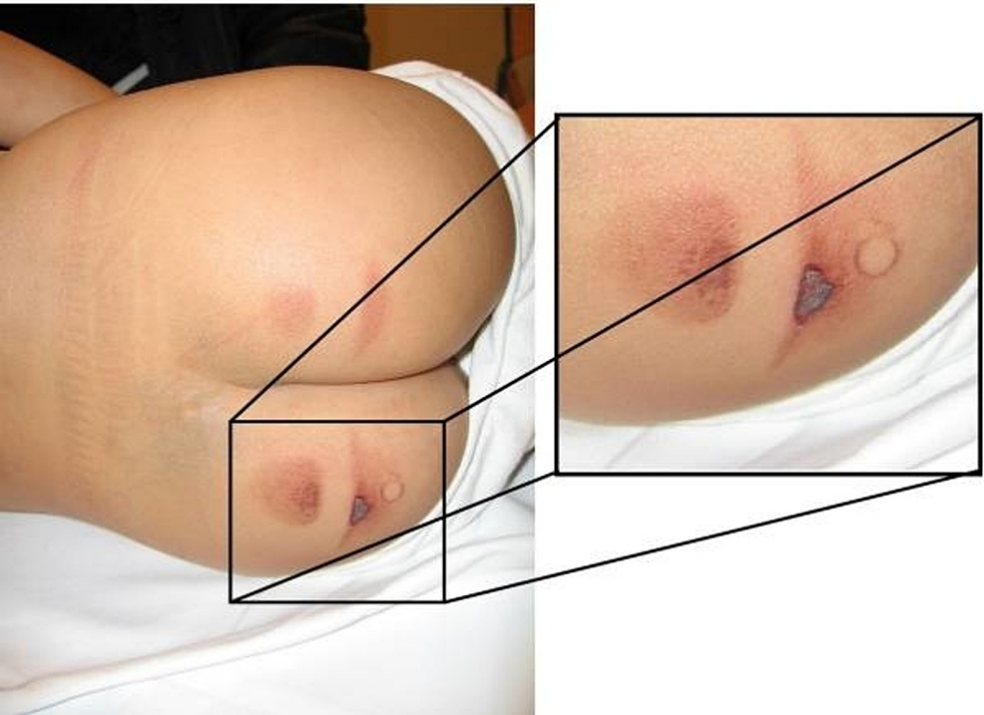
A four-year-old boy with Mongolian spots and bilateral sharply demarcated skin burns on the buttocks Well-defined imprint of an object (possibly an iron) can be seen. Case courtesy of Teeuw et al. [64]. (Creative Commons Attribution Non-Commercial License)

Abusive thermal injuries, inflicted by means of a dry source, mostly contact burns and retain the shape of the scorching object such as cigarette burns, i.e., characteristically reported as having a punched appearance [63], and iron marks (Figure 5) [64]. Dry thermal injuries on the back of the hand, forearm, buttocks, and back, should more frequently escalate the suspicion of abuse [63]. Dry thermal injuries may present as a classically isolated contact burn on the palms or feet, while nonaccidental burns are frequently multiple and patterned, therefore, timely documentation using pictures may even assist in identifying the object used to inflict the injury.

Similar to what has been previously narrated, a retrospective study analyzing a sample of over 15 thousand pediatric patients concluded a significant association between scald injuries with abuse and greater burnt body surface area [65]. Furthermore, Maguire et al. recognized intentional scalds as well-demarcated lesions affecting commonly the buttocks, perineum, or limbs [66]. Association between abusive burns and other injuries of abuse was also observed. On the other hand, accidental scalds mostly affected the upper body and had an uneven depth of injury (deepest at the point of first contact) with irregular borders [66]. Maguire et al. have further described the mechanism of accidental scald injuries as “pull over scald,” which includes burning injuries to the upper torso, upper extremities, head, and neck [67]. Younger children may frequently pull hot liquids down over themselves from a kitchen table and present with “spill and splash injuries” which are asymmetrical in 80% of the cases and more frequently on the anterior half of the body [67]. The location where the event of injury occurred has also been investigated as an indicator of abusive burn. In a study by Rosado et al., there was a significant association between burns occurring in the kitchen and accidental etiology [68].

Other Cutaneous Manifestations

Sentinel injuries are minor, unexpected, self-resolving, non-occult insults that do not require treatment, but are considered tocsins for abuse in pre-ambulatory children. Minor cutaneous lesions excluding accidental abrasions sustained during routine child care activities, and intraoral injuries such as bleeding, contusions, and tears, are considered sentinel for abuse [69]. A case-control retrospective study determined that 27.5% of the abuse victims had sentinel injuries in contrast to none of the non-abused children, and bruising was the most common sentinel injury followed by intraoral manifestations [43]. Patients with sentinel injuries require prompt initiation of investigations to uncover further occult injuries such as fractured ribs. Injury and abuse surveillance is indicated if explicit and comprehensible history following the trauma cannot be provided, or if the bruise is present on the nonbony surface of the body [69]. Usually, sentinel injuries are first documented by the primary care physician as an incidental finding during a physical examination and, as previously mentioned, do not require any specific treatment, but timely investigation and recognition may prevent serious abusive injuries in the future [69].

The presence of teeth marks should always be considered suspicious; that deems it crucial to recognize the difference between animal and human bite marks. Animal bites occur most commonly in children and have a variable canine distribution; on the other hand, human bites have a definite inter-canine length and are generally not associated with resultant skin tears [70]. Inter-canine length, measured by analyzing the bite mark, can also be useful in identifying whether the bite was inflicted by a child or an adult. Distance <3cm indicates that the human bite was inflicted either by a child or a small adult. Conversely, a distance >3cm indicates that the bite may have been caused by an adult [70]. Interestingly, the physicians should stay mindful of the fact that bite marks are currently the only lesion of abuse that can potentially serve to provide us with the identity of the perpetrator in situations where physical child abuse is suspected. DNA analysis and examination of the dental characteristics can be of aid in this regard [70].

Furthermore, traumatic hair loss secondary to mechanical twisting and pulling of the hair may also be reported in patients suffering from abuse [71]. It is worth noting that due to similarity in causative mechanisms, the abusive pulling of hair may be perceived as trichotillomania [71]. Scalp tenderness is occasionally observed in these patients.

Radiological manifestations

Radiologists are oftentimes the first to discover unexplained fractures and hence, play a pivotal role in diagnosing NAT. John Caffey, an American radiologist, had a keen sense of perception toward the multiple skeletal lesions present in children enduring chronic subdural hematoma (SDH). In all six of his cases, he noticed the lack of an unambiguous history of trauma to the head and the long bones [72]. These findings suggested perceptible inconsistencies in the accounts provided by the parents and the findings obtained on examination. Furthermore, John Caffey’s contributions also assisted in imparting an exceptional understanding of the psychological processes operating behind these inconsistent radiological lesions. Prior to Caffey, Ambroise Tardieu was the first to comprehensively describe various forms of child maltreatment [73], and his work “Forensic study on cruelty and the ill-treatment of children,” published in 1860, is described to be the first principle monograph examining various lesions identified in a battered child [74].

Radiology provides essential medical imaging procedures that aid in uncovering various concealed non-cutaneous lesions in a patient of suspected abuse, and these radiological findings should be explained in view of psychosocial history and reporting should focus on addressing anomalies, differential diagnoses, adequacy of imaging, and appropriate communication of NAT suspicion. Radiologists can additionally provide opinions on the credibility of the radiographs by identifying important landmarks and make comments on the suggestive mechanism of injury [75]. Radiological lesions of abuse can be broadly classified into three categories: osseous, visceral, and traumatic head injury due to child maltreatment (THI-CM), also known as abusive head trauma (AHT). Osseous lesions are the second most frequently detected signs of nonaccidental trauma [76]; and a skeletal survey (Table 1) [77], is the initial imaging protocol carried out in cases of suspected abuse to attain a primary image of any unrevealed fractures. Across the globe, the skeletal survey is considered the gold standard investigation in cases of suspected NAT, and it is inarguably fundamental in the diagnosis of skeletal injuries of abuse. Generally, it consists of several radiographic images covering the axial and appendicular skeletons of the body with the purpose of uncovering abusive fractures; however, the age of the patient, and determinants including the ability of the patient to verbally communicate their injuries, need to be considered to ascertain the utilization of this procedure. A skeletal survey is warranted in suspected cases of physical abuse in children under two years of age, and children aged between two and five years do not generally require a skeletal survey unless there is a suspicious lesion, evidence of fracture, or the patient is unable to verbally communicate a reliable history; possibly due to developmental delay [78,79].

Among the available protocols for skeletal surveys, the American College of Radiology-Society for Pediatric Radiology (ACR-SPR) survey [77], and The Society and College of Radiographers-The Royal College of Radiologists (SCoR-RCR) [80], survey is fairly accepted protocols and used internationally in various healthcare systems [81]. As per the SCoR-RCR protocol, a follow-up skeletal survey containing only a fraction of the original views, should ideally be completed within 11-14 days [81], of initial imaging, to potentially uncover further injuries or to determine the age of the fractures [78]. A delay of more than 28 days may result in the need to perform a complete skeletal survey instead of a follow-up one [80]. Radiographs of the skull, spine, and pelvis may be excluded from the follow up survey and have shown to reduce the radiation exposure of a follow-up survey [78,82]. Follow-up survey should cover areas of suspicion, AP chest with obliques, isolated AP views of the humerus and forearm or AP view of the entire arm (if possible), AP lower limb (hip to ankle) are indicated where possible but for larger children isolated AP femur and AP tibia and fibula should be carried out [80].

**Table 1:** ACR-SPR and SCoR-RCR skeletal survey parameters *Consider separate views in children older than one year †Consider separate views in larger children when the whole upper limb is not possible. AP humerus including shoulder and elbow, AP forearm including wrist and elbow, separate coned lateral views of elbow and wrist, and DP hand and wrist ‡In larger children when the entire lower limb is not possible, consider separate AP views of femur, tibia and fibula, knee, and ankle, along with separate coned lateral views of the knee, and ankle followed by DP foot. AP - Anteroposterior, PA - Posteroanterior, DP - Dorsoplantar

	ACR-SPR		SCoR-RCR	
	Structure	View	Structure	View
Axial Skeleton	Thorax with sternum, ribs, and thoracic and upper lumbar spine	AP, Lateral, Obliques	Chest including shoulders	AP, obliques to include all ribs
	Abdomen and pelvis with thoracolumbar spine and sacrum	AP	Abdomen and pelvis	AP
	Lumbosacral spine	Lateral	Entire spine*	Lateral
	Skull with cervical spine	Frontal, Lateral and Towne (if needed)	Skull	AP and lateral
Appendicular Skeleton	Humeri	AP	Whole upper limb (where possible) †	AP centered at elbow
	Forearms	AP	Elbow	Coned lateral
	Hands	PA	Hand and wrist	PA, and Coned Lateral (Wrist)
	Femurs	AP	Hip to ankle (whole lower limb, where possible) ‡	AP
	Tibiae and Fibulae	AP	Knee and ankle	Coned lateral
	Feet	AP	Feet	DP, and coned AP mortise view (Ankle)

**Figure 6:**
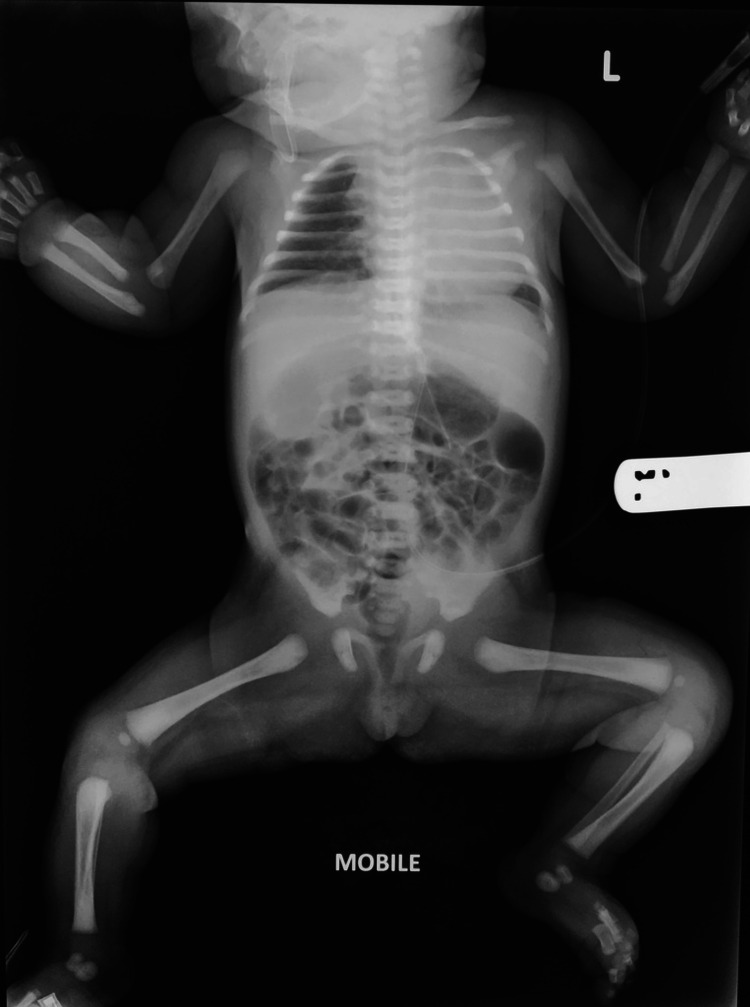
Normal babygram Case courtesy of Dr. Ian Bickle, Radiopaedia.org, rID: 54012

The effectiveness of follow-up skeletal survey is often subject to doubt since it may unnecessarily expose the patient to excessive radiation with inefficient utilization of healthcare and financial resources [83], but a single skeletal survey can potentially fail to uncover about 20% of the fractures [84]. Data have consistently highlighted the ability to follow up skeletal surveys to identify new fractures; however, the safety of the child should be of utmost value between these surveys [84]. Recommendations should be further evaluated to study the practicality of excluding pelvic and spinal areas in the skeletal survey since such injuries are uncommon in cases of NAT [83] but can be seen in cases of sexual abuse (especially fractures of the pubic ramus) [84]. Therefore, a balance between the risk of radiation exposure and child protection should be keenly considered, and focus should be maintained on developing elaborate protocols to minimize negative skeletal surveys [85]. Quality of the skeletal survey must be ensured and radiographs containing larger field views covering extensive sections of the skeleton must be avoided; therefore, it may be argued that a “babygram” (Figure 6) has limited utility in cases where physical child abuse is suspected [79]. However, the utility of a skeletal survey is not only limited to the victim of abuse, and contacts living under similar circumstances may also need to be evaluated [86].

**Figure 7:**
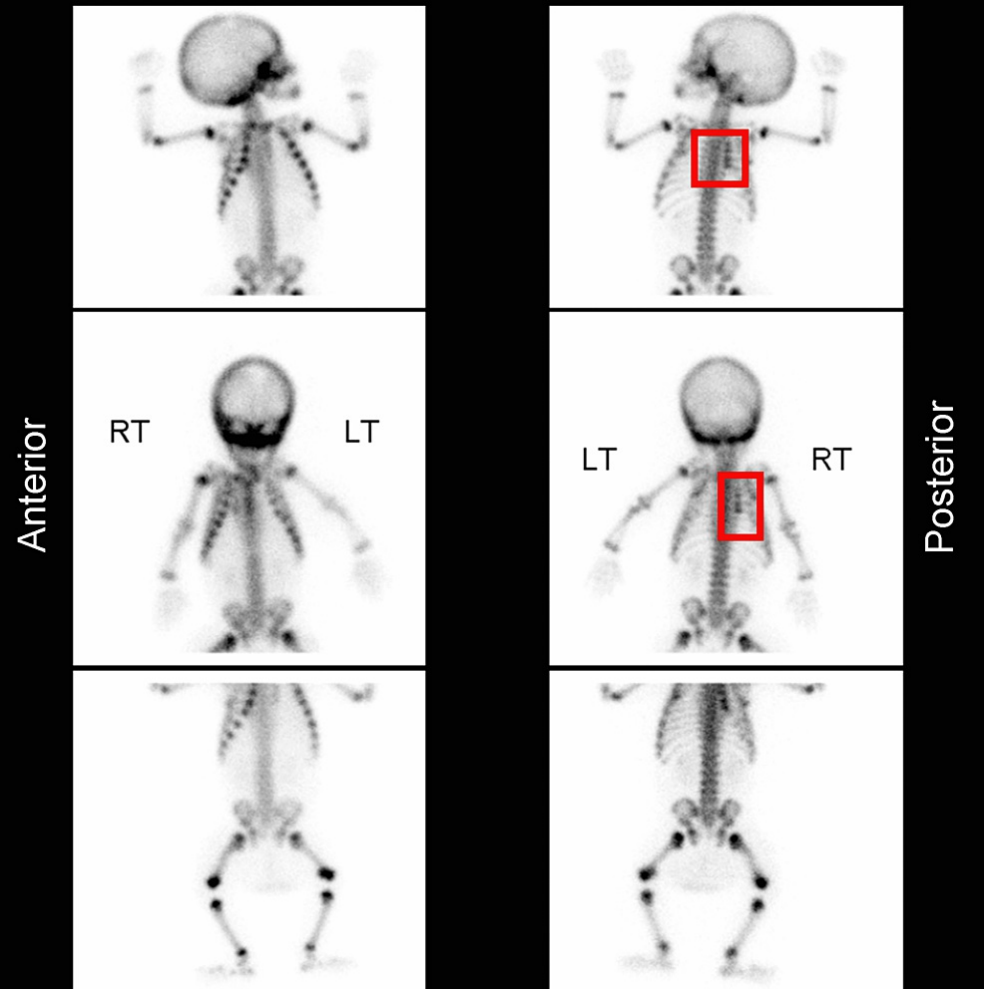
Radionuclide bone scan indicative of nonaccidental injury Focal increased uptake in the right fourth to seventh ribs posteriorly consistent with fractures. Case courtesy of Dr. Andrew Dixon, Radiopaedia.org, rID: 10321

In certain cases, the initial skeletal survey may find limitations in picking up nondisplaced fractures [87], however, such fractures can be highlighted in a follow-up survey. Considering the importance of timely intervention to safeguard the victims of abuse, follow-up survey may not be the most reasonable approach to detect these challenging fractures. Additional modalities such as ultrasound, CT scan, MRI, and nuclear bone scans can therefore be utilized in situations where diagnoses seem difficult. The skeletal survey additionally has low sensitivity for the detection of rib fractures which are one of the most common findings in cases of physical child abuse. Nuclear imaging modalities may be utilized in addition to the traditional skeletal survey but isolated use of these nuclear bone scans (Figure 7), appears to be of limited importance [88]. Two types of such imaging modalities are commonly used, Technetium-99m methylene diphosphonate (MDP) scan and positron emission tomography (PET) scan using Fluorine-18 labeled sodium fluoride. Both scans have decreased sensitivity to elucidate skull fractures, and classic metaphyseal lesions (CML) when compared to the traditional skeletal survey, however, the PET scan is more sensitive to posterior rib fractures and hence, can be used effectively to complement the skeletal survey [88]. Even further, the use of these imaging techniques is generally not indicated unless the skeletal survey proves to be ineffective despite high medical premonition of abuse [78]. Cost, lack of expertise, greater exposure to radiation, use of radionuclides, and inability to effectively pick up injuries in the first 72 hours [89], further limit the use of bone scans. The radiation dose of bone scans is considerably high compared to skeletal surveys; the dose equivalent to background radiation is approximately one year for bone scans versus equivalent to one month for skeletal surveys [90]. Despite these limitations, bone scans are considered an efficient replacement for a follow-up skeletal survey [84].

Non-contrast computed tomography (CT) can help in the diagnosis of THI-CM/AHT during the acute presentation. In particular, it can identify hemorrhages, skull fractures, and soft tissue swellings [91]. Therefore, a CT scan is indicated in patients with THI-CM/AHT regardless of the absence of neurological signs. Ultrasonography should only be performed as an adjunct to CT or MRI, as it has the advantage of being able to detect subcortical tears but falls short in the ability to pick up common CT detectable lesions observed as a consequence of traumatic injury to the head [91]. MRI is highly important in the evaluation of subacute, and old injuries; therefore, it is indicated for the assessment of non-acute cases of head trauma [85]. Overall evaluation and detection of parenchymal injuries, brain edema, contusions, and intracranial hemorrhages (ICHs) can be effectively carried out using MRI. Additional MRI may be indicated after an abnormal CT scan to carry out a prognostic evaluation of the child in presence of continuing neurological symptoms [85]. However, in cases of head trauma where an initial CT is unremarkable, MRI is indicated if the child exhibits continuing neurological symptoms. The utilization of whole-body MRI (WBMRI) in detecting occult osseous lesions of abuse is of limited importance. The need for sedation and reduced sensitivity to detect the fracture line indicates the inability of WBMRIs to replace routine imaging modalities but they can be used as an adjunct to direct targeted imaging [92].

**Table 2:** Comparison of various imaging modalities in cases of physical child abuse

Imaging Modality	Advantages	Disadvantages
Skeletal Survey	Gold standard investigation in detecting skeletal injuries of abuse. High quality images can help uncover undetected fractures and skeletal survey is a highly valuable modality in patients under 2 years of age and patients unable to communicate their injuries	Radiation exposure is one of the primary disadvantages of this imaging modality. Additionally, it needs to be supported by a follow up survey. It has limited utility in detecting nondisplaced fractures, and rib fractures. it is also technically demanding and appropriate immobilization of the patient is important.
Follow-up Survey	It can identify new undetected fractures such as rib fractures and Classic metaphyseal lesions (CML). It can also visualize nondisplaced fractures due to healing changes	Time is of utmost importance for a follow-up survey and safety of the patient needs to be ensured in between surveys. Excessive radiation exposure, inefficient utilization of financial and healthcare resources are also some its other drawbacks. Additionally, fractures may heal and become undetectable
Radionuclide Bone Scans	It is an efficient replacement for a follow-up survey and is more sensitive than a skeletal survey in detecting rib fractures, acromion fractures, and fractures of the spine. It can appropriately complement a skeletal survey.	Isolated use is not recommended in cases of suspected physical abuse. Limited utility in detecting skull fractures and CMLs. It is also technically demanding and radiation exposure is one of its primary drawbacks. Additionally, it can be falsely negative in the first 72 hours. It cannot additionally be used to date fractures. Not cost effective. Require sedation
Computed Tomography	Computed Tomography (CT) Scan of chest can detect rib and vertebral fractures. CT Chest has lower radiation exposure compared to a four-view chest radiograph. Contrast is not required unless visceral trauma is suspected. It is also indicated in patients with suspected THI-CM/AHT regardless of neurological signs. It can identify skull fractures, hemorrhages, soft tissue swellings in acute presentation. It is more sensitive than MRI in detecting acute intracranial hemorrhages. It is the investigation of choice in children with neurological findings. It can detect lesions requiring emergency intervention. Sedation is not required.	Contrast may need to be used particularly in cases of suspected visceral injuries; therefore, it is contraindicated in patients of renal failure and those in shock.
Magnetic Resonance Imaging (MRI)	Important in non-acute cases of inflicted head trauma. It is the most important imaging technique to comprehensively assess intracranial lesions. Whole body MRI (WBMRI) can also detect soft tissue injuries	It has limitations in acute setting. WBMRI is not recommended in the evaluation of skeletal injuries
Ultrasonography	Increased sensitivity for the detection of subcortical tears. It is a bedside imaging technique. It can help differentiate between various types of extra-axial fluids. It is additionally effective in detection of transphyseal and rib fractures when radiographic findings are negative. It can also pick up CMLs	It should not be performed as an isolated modality in cases of head injury. It cannot detect common lesions that a CT scan can detect, cannot detect several acute intracranial injuries including small subdural hemorrhages

Skeletal Lesions

Unexplained fractures are important radiological findings in a victim of child abuse. Skeletal lesions are particularly important in diagnosing abuse when the victim lacks the ability to effectively communicate information regarding the causative elements of their injury. Some fractures are more specific than others in hinting toward an abusive etiology [93]. CML, rib fractures, scapular fractures, fractures of the spinous process, and sternal fractures are considered highly specific for abuse.

**Figure 8:**
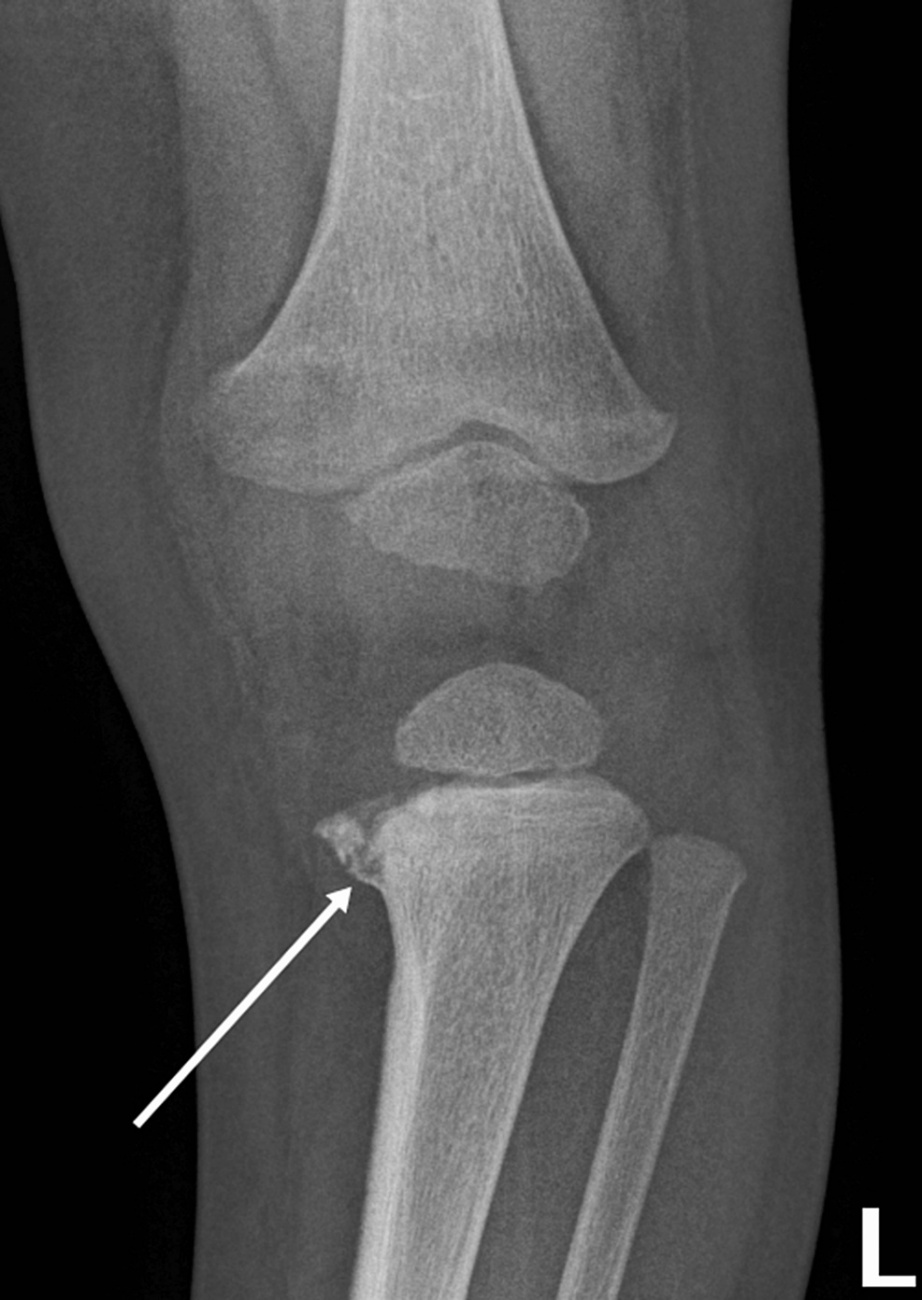
Bucket handle fracture (classical metaphyseal lesion) Case courtesy of Dr. Hani Makky Al Salam, Radiopaedia.org, rID: 13614

**Figure 9:**
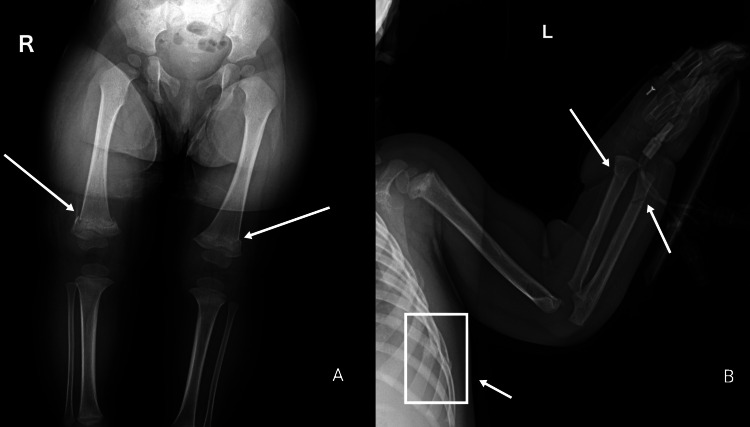
Rib fractures, bilateral classical metaphyseal lesions, and forearm fractures concerning for abuse Multiple fractures as outlined below: The left arm radiograph shows a buckle fracture of the distal radius, oblique fracture through distal ulnar diaphysis (the rib fractures are also visible on this radiograph) The lower limb radiograph shows metaphyseal corner fractures of the distal femurs Case courtesy of Rad_doc, Radiopaedia.org, rID: 47998

CML are highly associated with abusive etiologies. Two radiological presentations of the same metaphyseal lesion have been described depending on the direction of the x-ray beam; corner fracture, and bucket handle fracture [94]. Although no specific injury is pathognomonic for child abuse [95], CML (Figures 8, 9A) is still considered to be a highly specific finding, particularly in infants [96]. CMLs being a challenge in radiology are often missed on a simple radiograph due to unapparent periosteal elevation and hemorrhage [94]. Coned lateral views of the joints can be used as an adjunct to coned frontal views to improving the sensitivity of a skeletal survey to detect CMLs by 30% [97]. The ability of CT-scan and ultrasonography to detect CMLs are yet to be studied exclusively but there have been reports suggesting potential shortcomings of these modalities as well as of WBMRI to detect metaphyseal lesions [92,98,99]. Apart from metaphyseal lesions, fractures of the posterior and lateral ribs in infants resulting from violent squeezing, are also highly associated with a nonaccidental etiology [100]. In children aged less than three years, identification of any rib fracture (Figure 9B), connected to an unverifiable circumstance of injury, immediately warrants investigation to uncover an abusive cause [101]. Fractures of the first rib require a considerable amount of force and hence are practically implicative of child abuse in the absence of a corresponding traumatic event [102]. CML and rib fractures despite their high abuse specificity, due to their rare association with birth-related fractures, can still implicate a challenge in the diagnosis of abuse [103,104]. Additionally, there have been reports in the literature indicating inertial force, harsh manipulation, and motor vehicle collisions causing CMLs, thus indicating the absence of one specific injury characteristic of abuse [105].

As mentioned already in the previous section, the initial skeletal survey finds its limitations in detecting rib fractures when compared with CT-scan, bone scans, and follow-up survey. Including oblique views of the chest may increase the sensitivity of the initial skeletal survey to detect rib fractures by 19%-22%, especially for areas difficult to visualize such as the paravertebral space [82]. Fractures of the costochondral junction are reported as having a comparable appearance to the bucket handle fracture and are not highlighted in the initial skeletal survey [106]. CT scan of the chest can effectively direct the physician towards these fractures; additionally, it has been reported that costochondral fractures may concurrently exist with other abdominal visceral injuries, which indicates the importance of initiating protocols to uncover such lethal manifestations of child abuse when costochondral fractures are identified [106].

**Figure 10:**
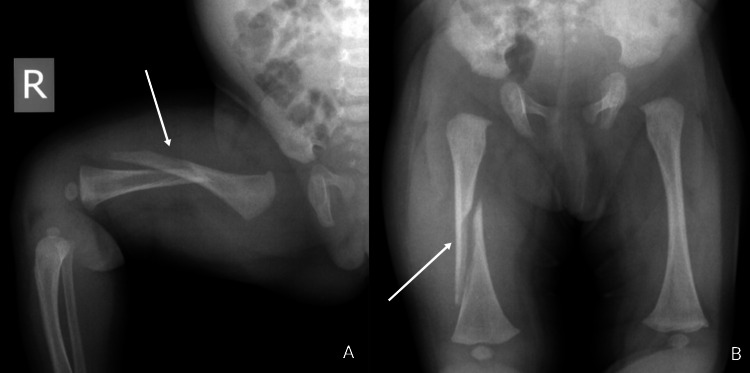
Spiral midshaft right femoral fracture suggestive of abuse Case courtesy of Dr Andrew Dixon, Radiopaedia.org, rID: 10321

Long bone injuries tend to present as spiral or oblique, and transverse fractures. Spiral fractures (Figures 10A, 10B), are particularly interesting for their atypical mechanism of development that is fairly unusual in a pre-ambulatory child [107]. Spiral fractures are generally the result of a torsional force, and unless there is a comprehensible explanation of the circumstance, these injuries prove to be rather indicative of abuse. It should be noted that spiral fractures of the tibia, termed “toddler’s fracture” is relatively frequent in ambulatory children with an incidence of 2.5 patients per 1,000 emergency cases in the United Kingdom [108]. Additionally, the general understanding among physicians remains that transverse fractures are less characteristic of abuse as compared to spiral or oblique fractures; however, it may also be argued that transverse long bone fractures are more prevalent than spiral or oblique fractures [109,110]. Multiple and bilateral long bone fractures in pre-ambulatory children are considered moderately specific for abuse (Figure 9B) [111].

Literature still lacks a detailed elaboration of the features of forearm fractures and their mechanisms of development which could provide physicians with a deeper understanding of the abusive or non-abusive circumstances of the injury. No specific forearm fracture is particularly associated with abuse, and these fractures are common in ambulatory children as a result of falling onto an outstretched arm; thus, proving to be a diagnostic challenge for healthcare professionals [112]. Buckle and transverse fractures were found to be the commonest forearm fractures in both abusive and non-abusive groups [112]. Isolated fracture of the ulnar shaft, known as Parry fracture, is speculated to be indicative of abuse but the results of a retrospective study were unable to significantly establish this association [113], so further researches are required to study this association in detail. An elaborate description of the mechanism of injury is indicated when such fractures are highlighted.

Although exceedingly significant, dating skeletal lesions prove to be a challenge in certain situations. It may seem essential for the radiologists to date how old a fracture is to compare this estimate with the account provided by the caregiver and to evaluate whether multiple fractures exist in various phases of healing, which could suggest repeated incidents of abusive trauma. Variation in the stages of healing observed in CML can make this process challenging. Sub-periosteal bone formation is an established sign observed in healing long bone fractures which may not be seen in CMLs [114]. However, bucket handle fractures observed in the acute phase are generally thinner as compared to those observed later in the healing stage [114]. Therefore, the aging of these fractures may not yield a significant advantage, and hence, a more patient-centered clinical approach would simply be to determine if the fractures are in different stages of healing [115]. This can provide information on the degree of a child’s safety by indicating risk for potential episodes in the future and has significant legal implications. Physicians should remain vigilant of the red flags associated with abuse such as unverifiable circumstances, fractures specific for abuse, the disproportionate association between swelling and fracture type, and fractures with a history of “fall” in children under one year [113].

**Table 3:** Summary points of some commonly seen fractures in cases of physical child abuse

Skeletal Lesions	Description
Classic Metaphyseal Lesions	These fractures are highly associated with abuse. Typically observed in the distal femur, tibia, and fibula, but can also be seen in proximal humerus, tibia, and fibula, and may be described as corner fracture, or bucket handle fracture. CMLs are particularly important in infants and may be missed on initial skeletal survey but can be detected on follow-up. WBMRIs, CT-Scans, sonographic imaging are not sensitive to CMLs
Rib Fractures	These fractures are highly specific for abuse. Mostly present as posterior or lateral rib injuries. Additionally, first rib fractures require significant force to develop and when present should raise suspicion. Rib fractures are difficult to detect on radiographs, especially if nondisplaced. Therefore, missed on initial skeletal survey but may be detected on a follow-up survey, CT-scan or nuclear bone scans
Costochondral Fractures	These fractures have a comparable appearance to bucket handle fractures and are difficult to detect on initial skeletal survey. Chest CT scan can help visualize these fractures effectively. Furthermore, these fractures may concurrently exist with abdominal visceral injuries and so warrant further investigation to uncover such occult trauma.
Long-bone Fractures	These can be transverse or oblique. Spiral or oblique fractures may be suggestive of a torsional mechanism of development. Additionally, long bone fractures are moderately specific for abuse. There is no specific forearm long bone fracture that is particularly associated with abuse
Skull Fractures	Complex, multiple, and diastatic skull fractures may hint towards abuse. Complex fractures alone do not provide sufficient evidence for an abusive etiology. Anatomical bone variations should be considered when analyzing skull fractures. CT scan has greater sensitivity than simple radiographs to detect skull fractures, particularly diastatic fractures.

Visceral Lesions

Abdominal injuries have a lower rate of occurrence as compared to other abusive lesions, but are categorized as the second most prevalent cause of mortality in physically abused children [116,117]. The severity of such injuries can be visualized by considering that 46% of the children presenting with nonaccidental abdominal injuries require emergency surgical procedures as opposed to only 5% of those presenting with other abusive lesions [118].

Contrast-enhanced ultrasound, and CT with IV contrast is the recommended imaging modalities for the detection of abdominal injuries. The difficulty associated with utilizing these imaging modalities should not be overlooked. Some visceral injuries may not be clinically evident and hence, prove to be a challenge for healthcare professionals [119]. This is of particular importance in younger children, and in patients having multiple distracting injuries. Therefore, it seems appropriate to utilize hepatic and pancreatic screening tests to uncover occult abdominal injuries. A cut-off value of 80 IU/L has been recommended for both transaminases, however, normal values do not rule out abusive abdominal injuries in children with high suspicion [120]; therefore, when negative, these tests may not be completely relied upon. Similarly, urinalysis could reveal occult insult to the renal system [121].

Various solid and hollow organ injuries can be identified in cases of physical abuse [122]. Essentially all abdominal viscera are at risk of being damaged as a result of trauma, and no specific abdominal injury is characteristically indicative of an abusive etiology; however, some abdominal injuries occur more commonly in cases of nonaccidental trauma. According to the results of a retrospective chart review, hollow visceral injuries were more commonly reported in physically abused children, and the presence of a solid organ injury in addition to a hollow visceral injury was only observed in cases of physical abuse [123]. On the other hand, solid organ injuries were observed as a result of both accidental and nonaccidental etiologies [123]. However, it is important to recognize that accidental abdominal insults are generally obvious with a coherent history of events such as road traffic accidents, and handlebar trauma.

Small bowel and pancreatic injuries are highly concerned for physical abuse. Hematomas, lacerations, strictures, and perforations may occur most commonly in the duodenum and proximal jejunum [124]. The retroperitoneal position of the duodenum provides it with a slight degree of protection against traumatic insults, therefore, a considerable degree of force is required for a duodenal hematoma to develop [125]. In cases of duodenal hematoma, the presentation may be suggestive of bowel obstruction, and the child may experience abdominal pain associated with obstructive vomiting [124]. In CT or barium upper gastrointestinal study, a submucosal mass may be evident which is replaced by a smooth or nodular fold thickening as the hematoma resolves [124]. Children with intestinal perforation mostly present with a fever and free intraperitoneal air may additionally be visualized on the radiograph [124]. Moreover, pancreatitis, pseudocysts, and intraabdominal abscess can also be detected on account of blunt force trauma in victims of abuse [126]. Chylous ascites associated with transected pancreas have also been reported in patients of inflicted trauma [127]. Therefore, the location of damage and its clinical presentation, when analyzed carefully, can contribute significantly to highlighting cases of abuse.

Adrenal lacerations are markers of extreme traumatic force and indicate an abusive etiology in the absence of a coherent history [128]. Similarly, if no consistent history of a vehicular collision or a bicycle handlebar accident is reported, the presence of pancreatic laceration in children under the age of five years is indicative of physical abuse [126]. Lacerations of the liver and lungs along with subpleural hemorrhage have also been detected in a battered child [129]. Cardiac trauma, although rare, may still be seen in cases of direct crush or blow injuries to the chest. Hemopericardium, contusions of the heart, aneurysms, and rupture have been reported in addition to the rupture of the thoracic duct [121].

Surgical consultation is necessary to appropriately manage patients enduring visceral injuries; a wide variety of solid visceral injuries can be managed non-surgically while surgical management is often proven to be necessary for the management of hollow visceral injuries [130-132]. Considering the high mortality associated with these injuries, apt delivery of suitable treatment is crucial; which if missed can result in enhanced severity of the injuries potentially proving to be fatal for the victim [132].

Traumatic Head Injury due to Child Maltreatment (THI-CM) or Abusive Head Trauma (AHT)

THI-CM/AHT can result in temporary or permanent neurologic impairment and is described to be the nonaccidental injury that most frequently results in lethal outcomes for the victim [133]. AHT, which is now referred to as THI-CM in Canada as revised in the Joint Statement on Traumatic Head Injury due to Child Maltreatment [134], is a severe type of pediatric physical abuse that typically involves injury to the cranium of a young child [135]. Intense shaking or application of a blunt force directly to the head can bring about varying degrees of neurologic impairment or even death in severe cases [135]. In fact, head injuries are the commonest reason behind traumatic death in children under two years [135], and so it may be argued that head trauma is the most severe form of physical child abuse. This type of injury usually begins with caretaker distress over a crying infant which leads to an aggressive episode of either intense shaking or direct head trauma. Shaking causes alternating deceleration and acceleration in the cranial vault and the mechanism of consequently resulting injury is called “Impulsive loading” [136]. Moreover, unlike impulsive loading injuries, the “Impact loading” insults result from a direct application of force to the head [136]. Therefore depending upon the mechanism of force that results in trauma, impulsive- and impact-loading injuries can also be termed dynamic and static injuries respectively [137].

The initial symptoms suggestive of THI-CM/AHT, such as increased sleeping, failure to thrive, poor feeding, vomiting, and lethargy, are somewhat nonspecific and only require supportive care [138]. However, some patients may present with more severe symptoms that require urgent care. These life-threatening symptoms include seizures, apnea, respiratory distress, shock, and bulging fontanelle [138].

**Figure 11:**
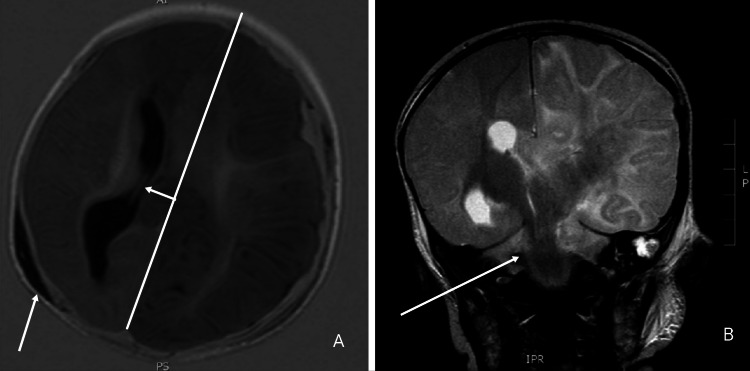
Subdural collection and herniation suggestive of THI-CM/AHT Selected T1 axial (A) and T2 coronal (B) images of the infant were performed three days after admission. Gross left cerebral edema with transtentorial and subfalcine herniation is shown along with the subdural collection. Case courtesy of Dr. Tony Lamont, Radiopaedia.org, rID: 17753

SDH (Figures 11A, 11B) is the most frequently observed injury in patients of THI-CM/AHT [136], and it is usually thin, diffused, unilateral, asymmetric, and rarely causes any significant pathological effects by displacing nearby structures [139,140]. The presence of SDH although moderately specific for THI-CM/AHT, may become more specific for abuse if an associated presence of retinal hemorrhages is established. Additionally, retinal hemorrhage as an isolated finding is moderately specific for abuse, but it can be a firm predictor if present as a bilateral, multilayered lesion that extends peripherally to the ora serrata on fundoscopy [141]. Diffuse parenchymal injury may also be seen along with SDH, but in cases of physical abuse, diffuse parenchymal injury is generally not an isolated finding except for in cases of strangulation [136]. Additionally, skull fractures should hint towards an abusive etiology when the fracture is complex, multiple, and diastatic [142], and complex skull fractures should not be used alone to make a diagnosis of abuse since these fractures can commonly be observed in cases of accidental trauma as well [143]. Furthermore, it is challenging to differentiate skull fractures from normal anatomical bone variations and accessory sutures on simple radiographs. CT-scan demonstrates a 10%-30% increased sensitivity in detecting skull fractures, especially diastatic fractures, when compared with a radiograph [82]. It is further unlikely that bone scans and MRI techniques be used for routine evaluation of skull fractures due to their increased rates of false negative results [82]. Sonographic evaluation of skull fractures has not been studied extensively but existing literature suggests a potential sensitivity of 91% for the detection of skull fractures when compared with a CT scan [82]. Ultrasonography may be falsely negative for linear fractures when the fracture is not directly underneath a hematoma [82]. Therefore, these are mostly utilized in evaluating areas of fracture suspicion or soft-tissue injuries [82].

In short, THI-CM/AHT may manifest as primary or secondary injuries [138]. Skull fracture, ICHs, and contusions are considered primary injuries resulting from direct traumatic insult, while several cellular inflammatory and molecular changes may give rise to secondary injuries as a consequence of complicated primary injuries. These secondary injuries may include cerebral edema, brain herniation, and stroke.

The psychological impact of nonaccidental trauma

Non-accidental trauma is associated with both physical and psychological impacts, in both the short and long term. Children who have experienced maltreatment may experience fear, distrust, isolation, and face difficulties with cognitive skills, attachment difficulties, behavioral challenges, and poor mental health outcomes [144]. The Adverse Childhood Experience (ACE) Study was one of the largest studies to investigate this relationship. Through a retrospective questionnaire given to thousands of adults regarding 10 different adverse events they may have experienced before the age of 18, including childhood abuse, neglect, and trauma in the child’s environment, they determined that the more ACEs an individual had experienced, their risk for depression, substance abuse, obesity, smoking, poor academic achievement, and early death considerably increased [145].

The effects of childhood trauma and physical abuse continue to be studied and those who have been abused have higher rates of suicide attempts [146], illicit drug use [147], and psychiatric disorders such as major depressive disorder, posttraumatic stress disorder, generalized anxiety disorder, panic disorder, ADHD, bipolar disorder, and substance use disorders [148-150]. Abuse and maltreatment can impair brain development and impact self-control, working memory, and cognitive flexibility [144,151], and can increase the risk for learning disability [152]. Children who experience maltreatment may also exhibit a range of behavioral disturbances such as anger, irritability, poor concentration, emotional withdrawal, intense emotional distress, and difficulty regulating emotions [153]. The psychological impacts of childhood maltreatment can have profound impacts starting in childhood, and continuing across the lifespan, contributing to significant morbidity and mortality.

Common mimickers of nonaccidental trauma

There are many medical conditions that may raise a false suspicion of child abuse, but a reasonable history must always be present to rule out an abusive intent. For instance, the presence of skin discoloration may raise a suspicion of abuse but such lesions can occur in several hematological and skin conditions as well; so, repeat examination and follow-up clinical visits should be deemed necessary in order to establish a firm diagnosis [154]. In general, clinical lesions that mimic nonaccidental trauma may belong to one of three broad categories: mimickers of cutaneous nonaccidental injuries, mimickers of abusive fractures, and mimickers of THI-CM/AHT. These may also be classified into essentially two main categories based on origin, genetic and acquired abnormalities.

Genetic and Congenital Disorders

Hemophilia and von Willebrand disease are two congenitally present mimickers of child abuse that have cutaneous lesions somewhat comparable to those observed in cases of nonaccidental trauma [155]. However, bruises of such congenital bleeding disorders are larger and more numerous in comparison to those caused by abuse [155]. Children with hemophilia are also at substantial risk for developing spontaneous ICH that can potentially be misperceived as an injury sustained during a violent abusive episode [155]. Such congenital bleeding disorders may not always be apparent on a single physical examination; therefore, it is crucial to document previous episodes of bleeding and bruising with difficulty in controlling surgical, and accidental bleeds. Detailed family history in such challenging cases can also uncover various hereditary bleeding disorders.

**Figure 12:**
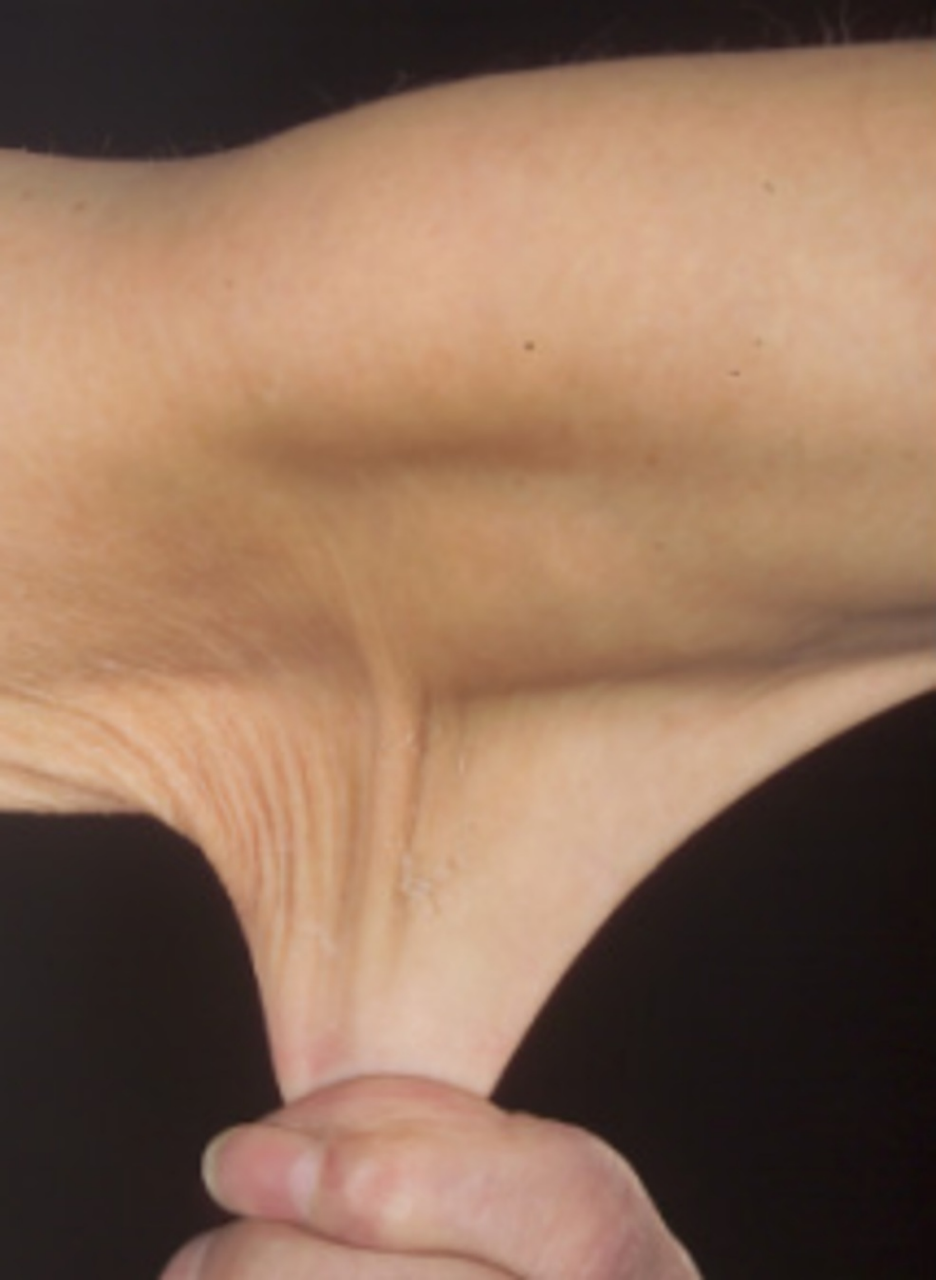
Hyperextensible skin associated with Ehlers-Danlos syndrome Case courtesy of JK Whitaker, P Alexander, DY Chau, NL Tint. Wikimedia Commons. Creative Commons Attribution 2.5 Generic license.

**Figure 13:**
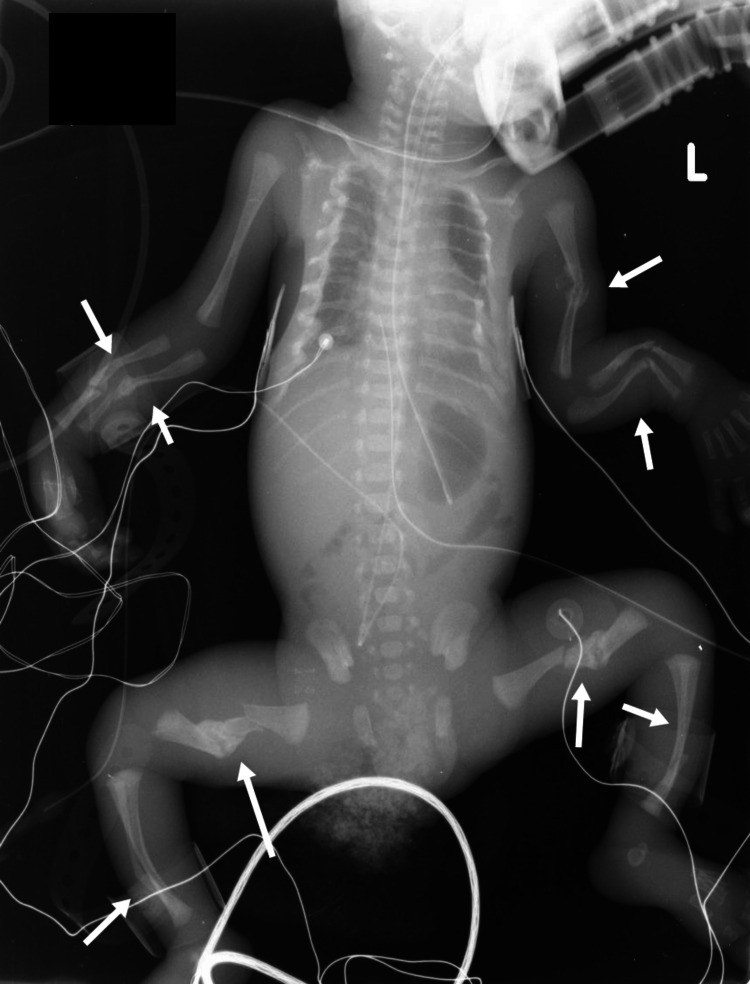
Osteogenesis imperfecta in a premature neonate Endotracheal tube and umbilical arterial and venous catheters were well placed. The patient is on a ventilator. Multiple fractures incurred in utero at various stages of healing; many with malunion, angulation, and bridging callus: multiple ribs bilaterally, left humerus, radii and ulnae, femora (segmental fractures in both), and fibulae. Case courtesy of Dr. Yair Glick, Radiopaedia.org, rID: 52436

**Figure 14:**
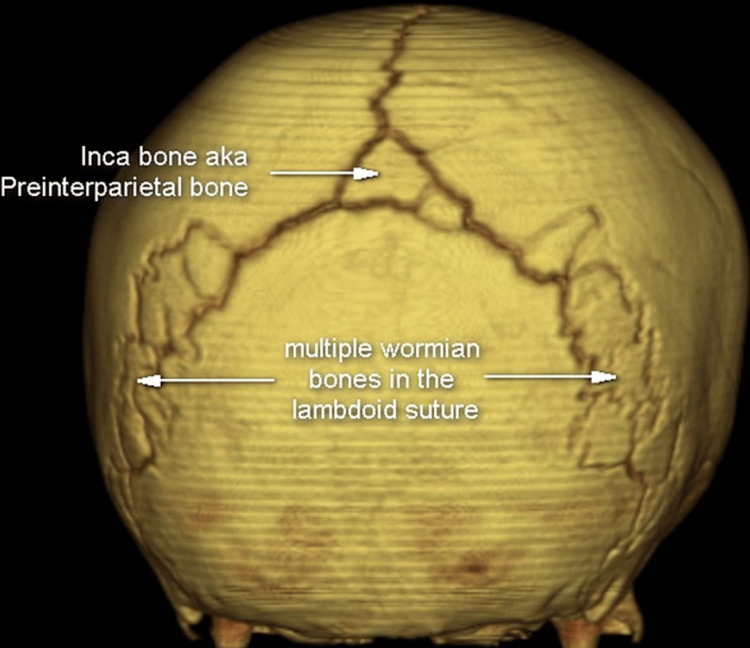
Wormian bone in lambdoid suture along with Inca bone Case courtesy of Assoc. Prof. Frank Gaillard, Radiopaedia.org, rID: 36312

**Figure 15:**
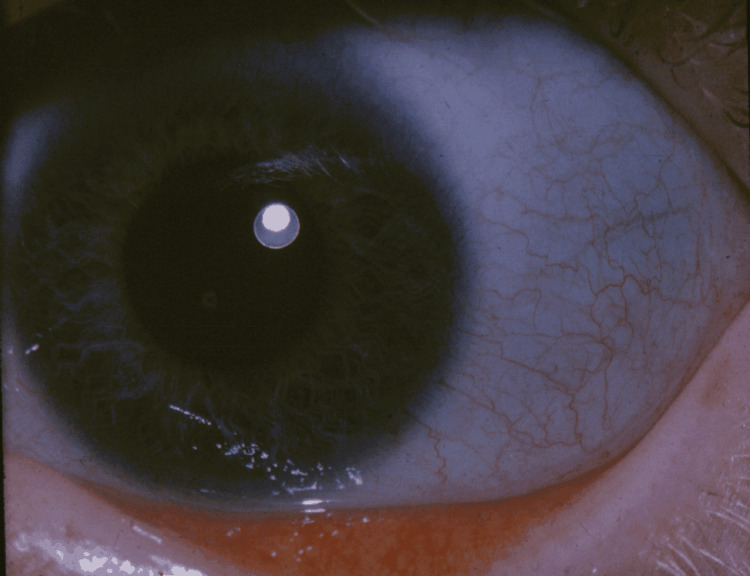
Blue sclera associated with osteogenesis imperfecta Case courtesy National Eye Institute (Public domain)

Genetic disorders of collagen synthesis, including osteogenesis imperfecta (OI) and Ehlers-Danlos syndrome (characterized by loose skin as shown in Figure 12, joint hypermobility with increased risk of dislocation, and susceptibility to bruising) may have similar skin lesions as well [155]. OI predisposes bones to fractures, as can rickets; however, the fractures reported as a result of abusive insults are generally not a consequence of either condition [155]. Diagnosis of OI is fairly apparent based on clinical, radiological (Figure 13), laboratory, and molecular (COL1A1, COL1A2, and IFITM5) findings, but these features are subject to high clinical variability, and cases lacking elaborate findings may be susceptible to misdiagnosis [155]; however, bowing of bones (Figure 13), presence of wormian bones (Figure 14), and osteopenia can help differentiate nonaccidental skeletal findings from OI (while other findings of OI such as dental involvement, and blue sclera [Figure 15], are equally important but may not always be present, especially in milder cases). In addition to bone abnormalities, poor vascular integrity can also be seen in disorders of collagen synthesis, which increases the risk for the development of a SDH, and hence, poses a challenge in the diagnosis of THI-CM/AHT [155]. Similarly, Arita et al. have also documented Menke’s disease with large bilateral subdural hematoma, and metaphyseal spurs at the lower ends of long bones without a significant family history of neurological diseases, as a differential diagnosis of THI-CM/AHT [156]. Similarly, infantile acute lymphoblastic leukemia, another genetic disease, leads to a SDH, retinal hemorrhage, and encephalopathy that can prove to be fatal and are highly suggestive of THI-CM/AHT [157].

Acquired Abnormalities

**Figure 16:**
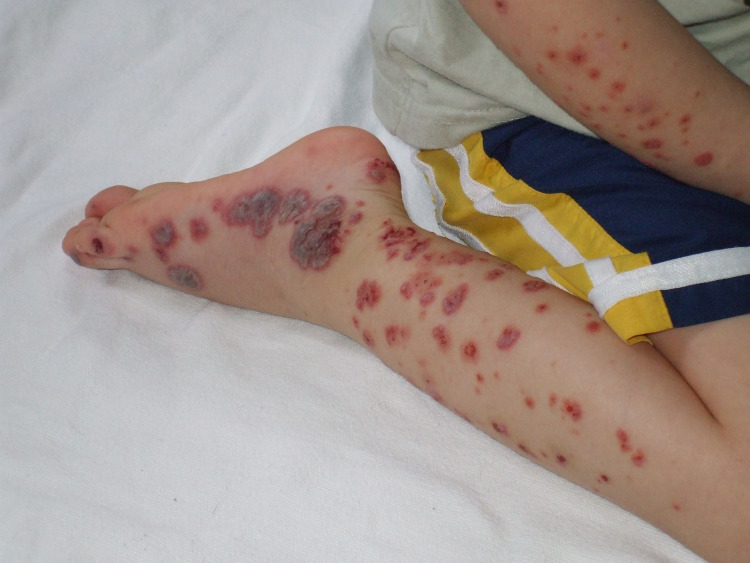
Henoch-Schönlein purpura (HSP) on the right leg of a child Wikimedia commons (Public domain)

**Figure 17:**
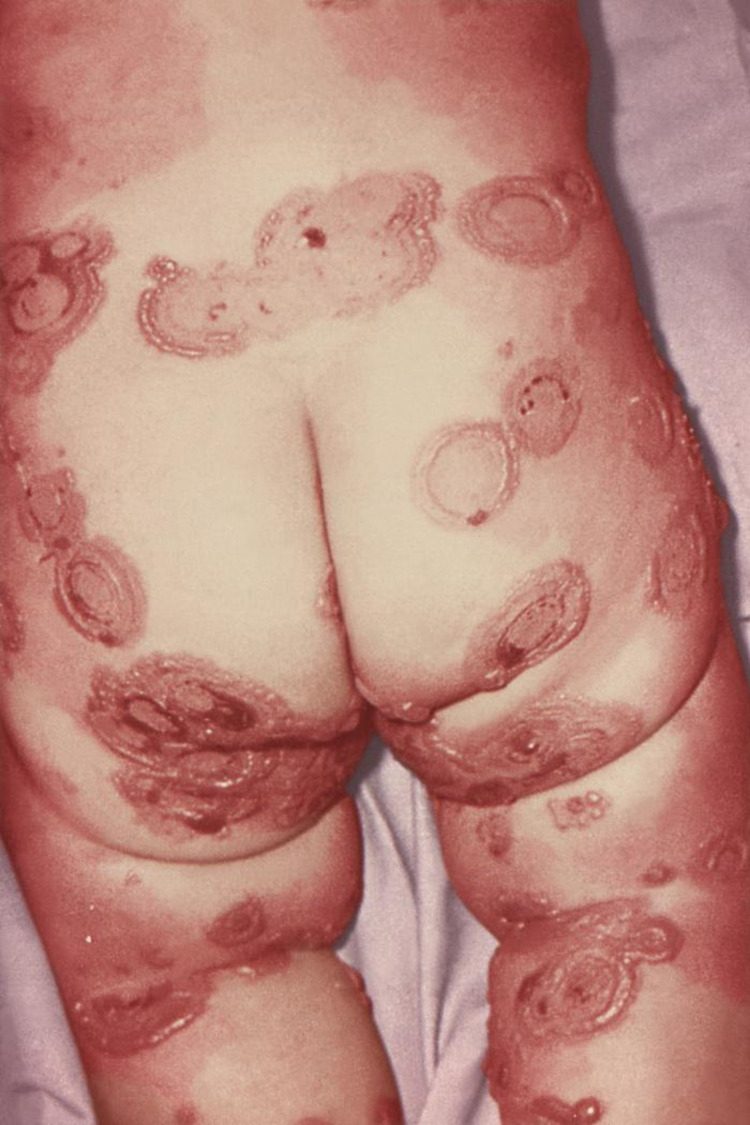
Classic bullous erythema multiforme lesions, known as Stevens-Johnson syndrome Case courtesy of CDC/Arthur E. Kaye. Public Health Image Library (PHIL).

**Figure 18:**
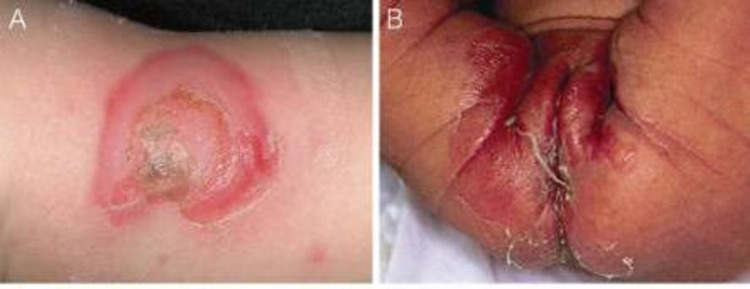
Staphylococcal skin infections (A) Bullous impetigo. (B) Staphylococcal scalded skin syndrome. Case courtesy Masayuki AMAGAI [159]. Creative Commons Attribution License

Dermal melanocytosis, also called Mongolian spots (Figure 5), largely seen in Black, Asian, and Latino ethnic groups may also be misperceived as bruises especially when present over the buttocks [158]. Such pigmented areas of skin do not rapidly progress in color and size, differentiating them from bruises. Henoch-Schonlein purpura (Figure 16), and erythema multiforme (Figure 17), can also be mistaken for nonaccidental bruising especially when the child is otherwise apparently healthy. Drug history of sulfonamide or penicillin use or past medical history of infections holds importance in diagnosing erythema multiforme [158]. Cutaneous Staphylococcal infections (Figure 18) [159], such as scalded skin syndrome, and bullous impetigo having erosions and blisters with shiny appearances can also be confused with abusive scalds.

**Figure 19:**
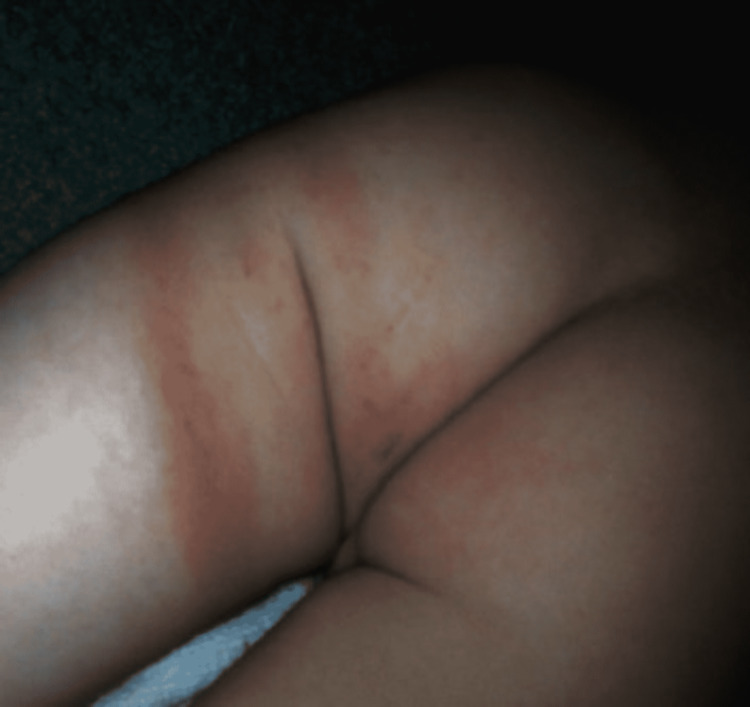
Erythema migrans mimicking abusive thigh bruising Case courtesy Pan et al. [160]. (Creative Commons Attribution License)

Similarly, a situation of NAT misdiagnosis can occur in the case of Lyme Disease; the typical bull’s-eye rash or erythema migrans seen in such a scenario can be mistaken for an abusive lesion (Figure 19) [160]. Langerhans cell histiocytosis is another acquired medical condition that presents signs such as skull fracture, erythema, and erosion in labia majora which can easily be misinterpreted as evidence of child maltreatment [161]. Additionally, certain dermatologic conditions such as phytophotodermatitis, caused by furocoumarin-containing plants, can lead to suspicions of abuse in the initial stages as reddish-brown lesions appear but their subsequent progressions with time do not match bruising thereby removing doubt [162]. Similarly, non-furocoumarin-containing chemicals may cause similar presentations that may mimic child abuse through a process called auto-oxidation as in the case of certain over-the-counter moisturizers that cause contact-dermatitis [163]. Berloque dermatitis is one such type of photo contact dermatitis caused by bergamot (often found in perfumed products) that leads to hyperpigmentation, erythema, and sometimes blistering [164].

The rising popularity of home births has also resulted in cases with signs mimicking that of child abuse. These results are primarily due to the fact that such infants are often not administered Vitamin K after birth making them vulnerable to deficiency, bleeding, and disseminated intravascular coagulopathy raising concern regarding the child’s wellbeing among the physicians [165]. Another vitamin deficiency, that of cholecalciferol, can play a similar role by causing detrimental effects on infants. Such effects can again be suggestive of child maltreatment and therefore present physicians with a worrisome scenario [166].

Additionally, a notable consideration in certain parts of the globe is trauma inflicted during traditional medical practices. Understanding these traditional healing practices local to a specific area is equally important in certain settings [158]. Dermabrasion practices, cheat sah in the Chinese culture and Cao gio in Southeast Asian practice, can leave lesions on the skin similar to those observed in cases of abuse [158]. Similarly, ecchymosis or uncommonly even burns observed during cupping therapy can be mistaken for physical abuse [167]. As highlighted by Lupariello et al., considering the similarity between the lesions, an initial suspicion of child abuse may distract the physicians and the case may become subjected to misdiagnosis; intervention of multidisciplinary and multispecialty teams, however, is necessary for such situations to establish clarity [167]. Therefore, it is important for all healthcare practitioners to be mindful of such practices in the relevant setting when dealing with cases of suspected abuse.

Miscellaneous

Quite often it is also possible that a neonate may present with lesions that are not associated with any medical condition and therefore, may give rise to suspicions regarding its treatment by caregivers. Such is the case of self-inflicted bruises on infants due to excessive, forceful sucking often seen on forearms [168]. Similarly, it has been reported that in cases of congenital penoscrotal webbing, vigorous cleaning of the genital region can lead to its avulsion and bleeding which may present as a sign of abuse [169].

The shaken baby syndrome is a term often encountered in the analysis of literature on child maltreatment. It is characterized by subdural hemorrhage, brain swelling, and retinal hemorrhages, the “classic triad” resulting from excessive acceleration and deceleration. Similar movements can also occur in non-abusive cases such as when a child sits on a rocking toy and experiences forces that result in injuries suggestive of abuse [170]. Further analysis of available literature has also brought forward examples that involve medical intervention that can be confused with abuse. For example, non-surgical treatment of clubfoot involves forced eversion and dorsiflexion, and subsequent casting. This manipulation can result in a metaphyseal injury similar to that seen in the case of indirect force on a limb - classic metaphyseal fracture (CML) [171].

Reporting nonaccidental trauma

Across many jurisdictions and nations, physicians have a mandatory duty to report suspected NAT. It is not the responsibility of the physician to determine the intent or if child abuse did in fact occur, but to act in the best interest of their patient to ensure safety and facilitate further investigations. Failure to report suspected cases can result in grave negative outcomes for the patient, and the physician can be held legally accountable in the form of persecution, fines, or imprisonment [172]. While guidelines for reporting can vary widely, in general, they are multi-disciplinary and start with a well-documented history of the injury, physical exam and skeletal survey, collateral history from caretakers, photographs and radiological evidence, review of previous documentation of injuries, and relevant lab work. They should ascertain if there are other children who could be involved or at risk. In some cases, the child may disclose the abuse themselves. Regardless of the circumstances, if NAT is suspected, the physician should act in a timely manner and keep the best interest of the patient in mind to prevent further harm [173]. When a report is deemed appropriate, jurisdiction guidelines should be consulted, and the appropriate child protection service agency should be contacted, or the police. In the USA, after a report is made, it becomes the responsibility of the child welfare organization to carry out further investigations. Most investigations do not result in the child being removed from the home and can connect families with additional supports such as individual and/or group therapy, parenting classes, home visits, and access to follow-up care [174]. In some instances, the legal system will become involved, and the child may be temporarily removed from the home [174].

There are many factors that can influence a physician’s decision to report, and even in cases of suspected NAT, they are not always reported. A study investigating pediatricians in an office-based study determined that 10% of all injuries were deemed to be suspicious, but only 6% were reported [175,176]. An article in the AMA Journal of Ethics highlighted the common themes from that study that influenced reporting; they included relationships to the family (familiarity and positive experiences made physicians less likely to report), case-specific elements, use of available resources (such as reviewing the case with a colleague), and past experiences with reporting [177].

## Conclusions

Nonaccidental trauma is a multidimensional issue that proves to be a significant challenge in routine healthcare; comprehensive education regarding NAT for medical providers at all stages of training may help to decrease barriers to identification and timely management of this issue. It is a preventable cause of significant mortality and morbidity in children, thus requiring physicians to maintain an appropriate index of suspicion when dealing with high-risk patients displaying red flag findings. Younger children, children with heightened health needs, prematurity, lack of social or family support systems, history of psychiatric illness in parents, drug use, rate of violent crimes and poverty in the neighborhood, and intergenerational abuse are some of the risk factors associated with child maltreatment. While efforts have been made previously to explore child abuse in a clinical setting, this review intends to build up a multispecialty approach to identifying and diagnosing NAT in children, therefore, discussing in detail, bruises, burns, fractures, abdominal, and intracranial injuries associated with abuse. Emphasis on careful interpretation, documentation, and reporting of these injuries in light of the circumstantial account and the psychosocial history can offer substantial proof to support the case. Special care must also be taken to acknowledge the victim’s post-trauma psychological status and thus provide any therapeutic support essential. Assessment of the existing literature has additionally brought forth the need to delve further into research surrounding specific nonaccidental injuries such as certain forearm fractures in addition to the effects of societal changes such as the impact of the COVID-19 pandemic on the pattern of NAT incidences.
